# Queensland Telepaediatric Service: A Review of the First 15 Years of Service

**DOI:** 10.3389/fdgth.2020.587452

**Published:** 2020-11-25

**Authors:** Anthony C. Smith, Nigel R. Armfield, Mark G. Coulthard, Michael L. Williams, Liam J. Caffery

**Affiliations:** ^1^Centre for Online Health, The University of Queensland, Brisbane, QLD, Australia; ^2^Centre for Health Services Research, The University of Queensland, Brisbane, QLD, Australia; ^3^Centre for Innovative Medical Technology, University of Southern Denmark, Odense, Denmark; ^4^Recover Injury Research Centre, The University of Queensland, Brisbane, QLD, Australia; ^5^Pediatric Intensive Care Unit, Queensland Children's Hospital, Brisbane, QLD, Australia; ^6^Mayne Academy of Pediatrics, Faculty of Medicine, The University of Queensland, Brisbane, QLD, Australia; ^7^Mackay Base Hospital, Queensland Health, Brisbane, QLD, Australia

**Keywords:** telehealth, telemedicine, telepaediatrics, digital health, indigenous, specialist health care, models of care, regional and remote health services

## Abstract

In November 2000, the Queensland Telepaediatric Service (QTS) was established in Brisbane, Australia, to support the delivery of telehealth services to patients and clinicians in regional and remote locations. The QTS was built on a centralized coordination model, where telehealth services could be effectively managed by a dedicated telehealth coordinator. In doing so, telehealth referral and consultation processes were efficient and clinicians felt better supported as they adjusted to new processes for engaging with patients. We have conducted a retrospective review of activity associated with the QTS and summarized key activities which have arisen from this extensive program of work. Telehealth service records and associated publications were used to describe the evolution of the QTS over a 15-year period. From November 2000 to March 2016, 23,054 telehealth consultations were delivered for 37 pediatric clinical specialties. The most common service areas included child and youth mental health, neurology, burns care, surgery, and ear nose and throat services. A range of different telehealth service models were developed to align with different clinical service needs and location of services. Whilst most work involved video consultation between hospitals, some services involved the delivery of telehealth services into the home, schools or community health centres. Despite its longevity, the QTS was not immune to the usual challenges associated with telehealth implementation, service redesign and sustainability. Experience reported from the QTS will be useful for other health services seeking to develop comprehensive telehealth services in a rapidly changing healthcare environment.

## Introduction

Conventional models of health care in Australia require patients to travel (often great distances) to receive specialist care. Occasionally specialist teams travel to remote communities to deliver health care services; but these tend to occur on an intermittent basis. For logistical reasons, some patients do not receive the care they require because of the difficulties of having to leave their community for an appointment and/or treatment. Telehealth can be used to improve access to health services for people living in distant locations; this is important in Australia where the majority of specialist health services are based in metropolitan areas, and the distances between these hospitals and small rural hospitals may be considerable.

Over the last two decades, the use of telehealth to deliver pediatric telehealth services (telepaediatrics) has been reported by many countries (1, 2). Telepaediatric service models have included multidisciplinary services operated from a centralized coordination centre; discipline-specific telehealth services for children and young people; and services in different settings (such as hospitals, community health settings, schools and in the home). The idea of providing telehealth services particularly for children and their families makes sense because of the centralization of pediatric specialist services, imposition of travel away from home, and the requirement for a child to be accompanied by a parent or caregiver for travel to and from their specialist appointment.

**Figure 1:**
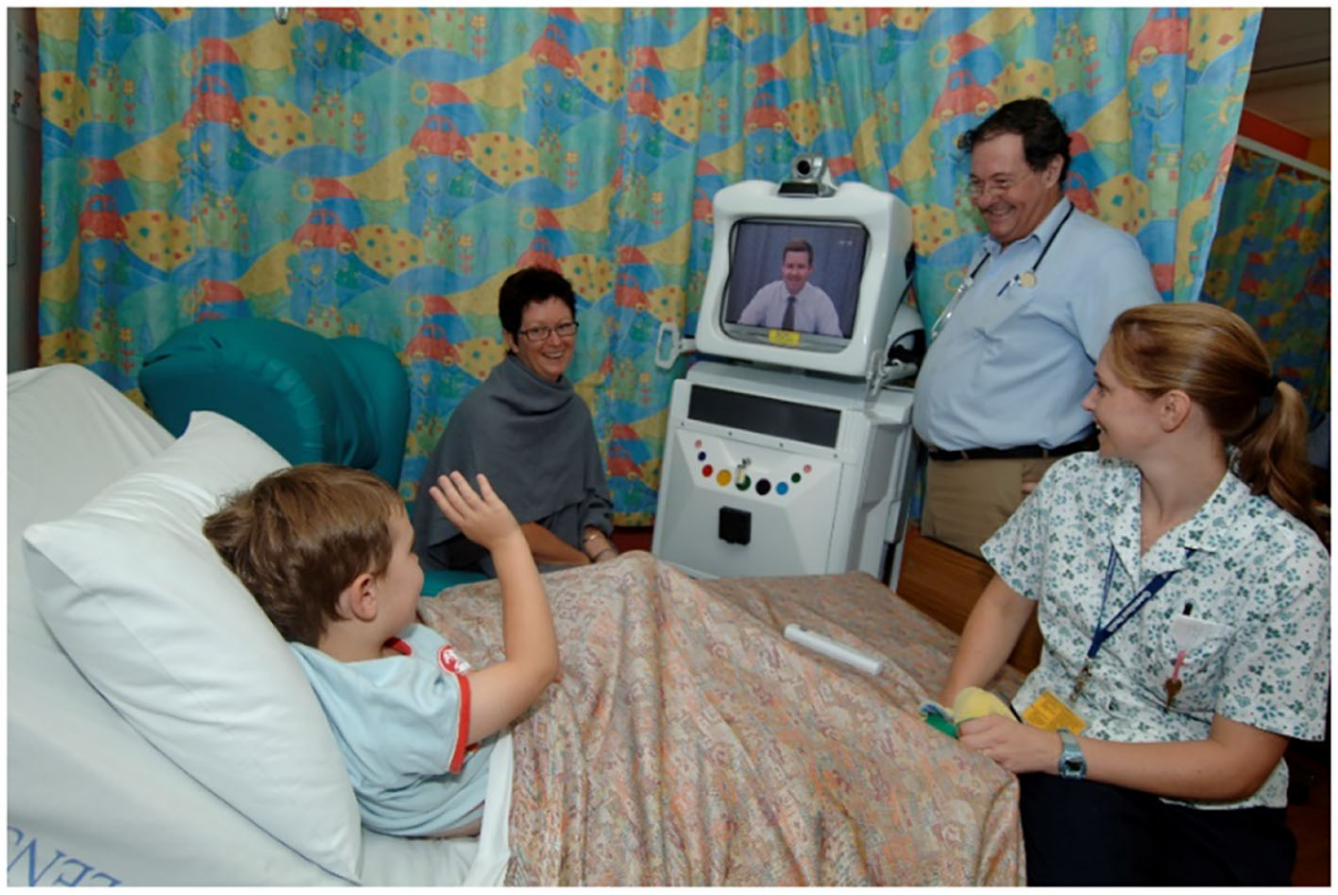
Wireless (robot) videoconference system used for bedside consultations in regional pediatric wards.

Despite the clear benefits of telehealth, a long-term effort was required to address the challenges of telehealth implementation and uptake in the Queensland public health service. In November 2000, a pediatric telehealth service model was established at the Royal Children's Hospital (RCH) in Queensland, Australia (3). The Queensland Telepaediatric Service (QTS) offered a convenient referral process (single point of contact) for telehealth referrals and coordination of telehealth consultations (4). The majority of telehealth consultations involved a videoconference appointment between the specialist hospital in Brisbane and a referring hospital. Other communication methods included correspondence by email or telephone. The telehealth service used the videoconferencing network operated by the state health department—comprising both hardware and software systems. In some cases, customized telehealth systems were deployed to improve child-friendliness, or where standard systems did not meet the clinical requirements, see Figure 1 (5).

The University of Queensland's Centre for Online Health (COH) was responsible for establishing and operating the QTS in partnership with the Queensland health department. Operational responsibilities were funded by a service level agreement; and an integrated research program was funded by community and corporate organizations. The aim of this review is to summarize patterns of service activity, outline specific service models, and describe the key enablers and challenges associated with the service.

## Methods

This study presents a retrospective review of QTS activity reported over a 15-year period from November 2000 to March 2016. Service activity was obtained from an operational database, which was owned and maintained by the COH. This database contained information about each consultation including specialty, duration, location and modality. This review also summaries published studies undertaken during the course of this program. All published studies reported in this review received ethical approval from the appropriate committees. Further permission and exemption from ethical review was obtained from Children's Health Queensland Hospital and Health Service Human Research Ethics Committee to publish overall service activity according to service records managed by the COH (dated 13 June 2019).

## Results

### Service Activity

**Figure 2:**
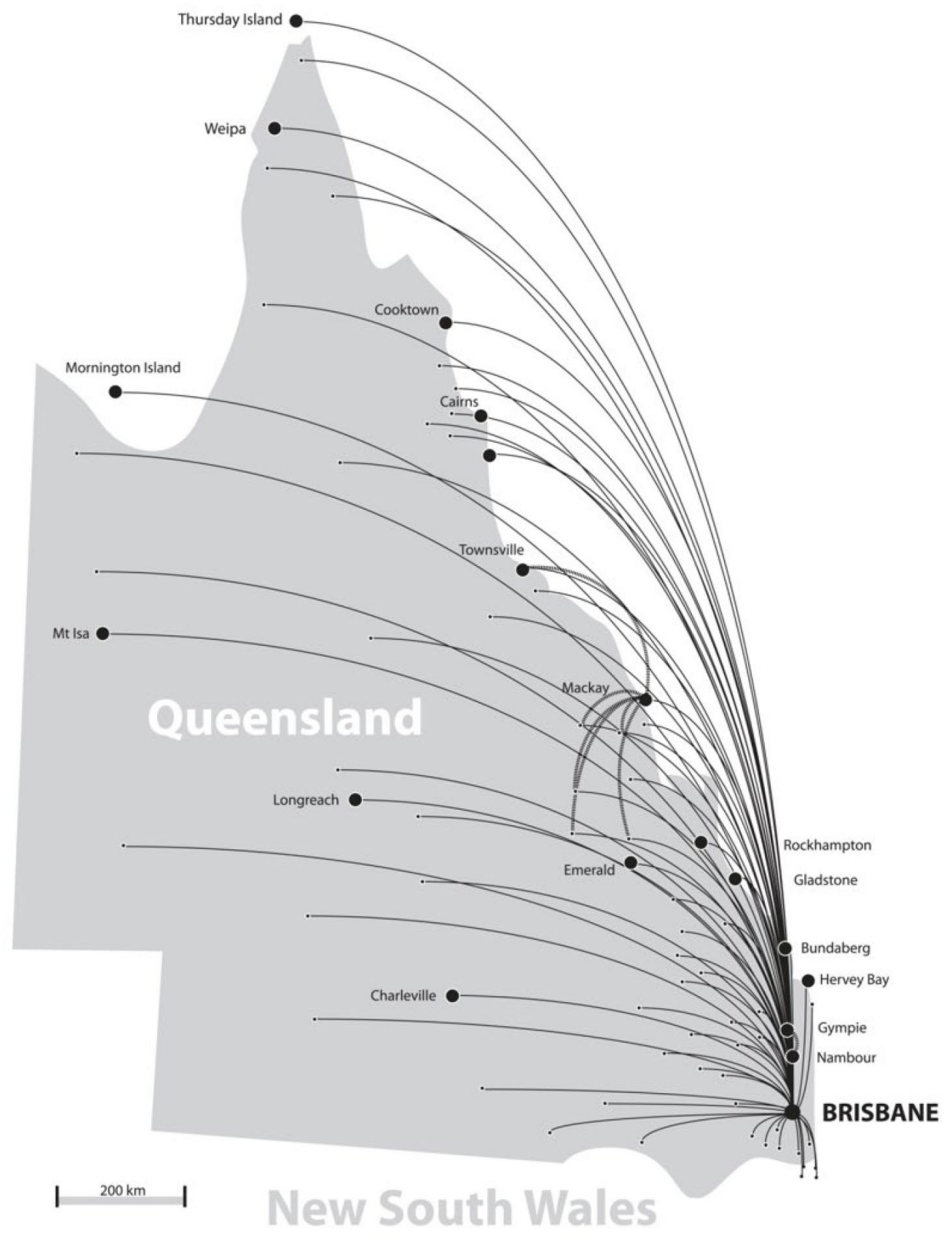
QTS main referral sites in Queensland and New South Wales.

From November 2000—April 2015, a total of 23,054 telehealth consultations were coordinated through the QTS. The majority of these (95%) involved consultations by videoconference, whereas the remaining involved email (3%) or telephone consultations (<1%). A total of 37 clinical specialties were actively involved in the QTS, delivering services to 110 sites throughout Queensland and Northern New South Wales, see Figure 2.

**Figure 3:**
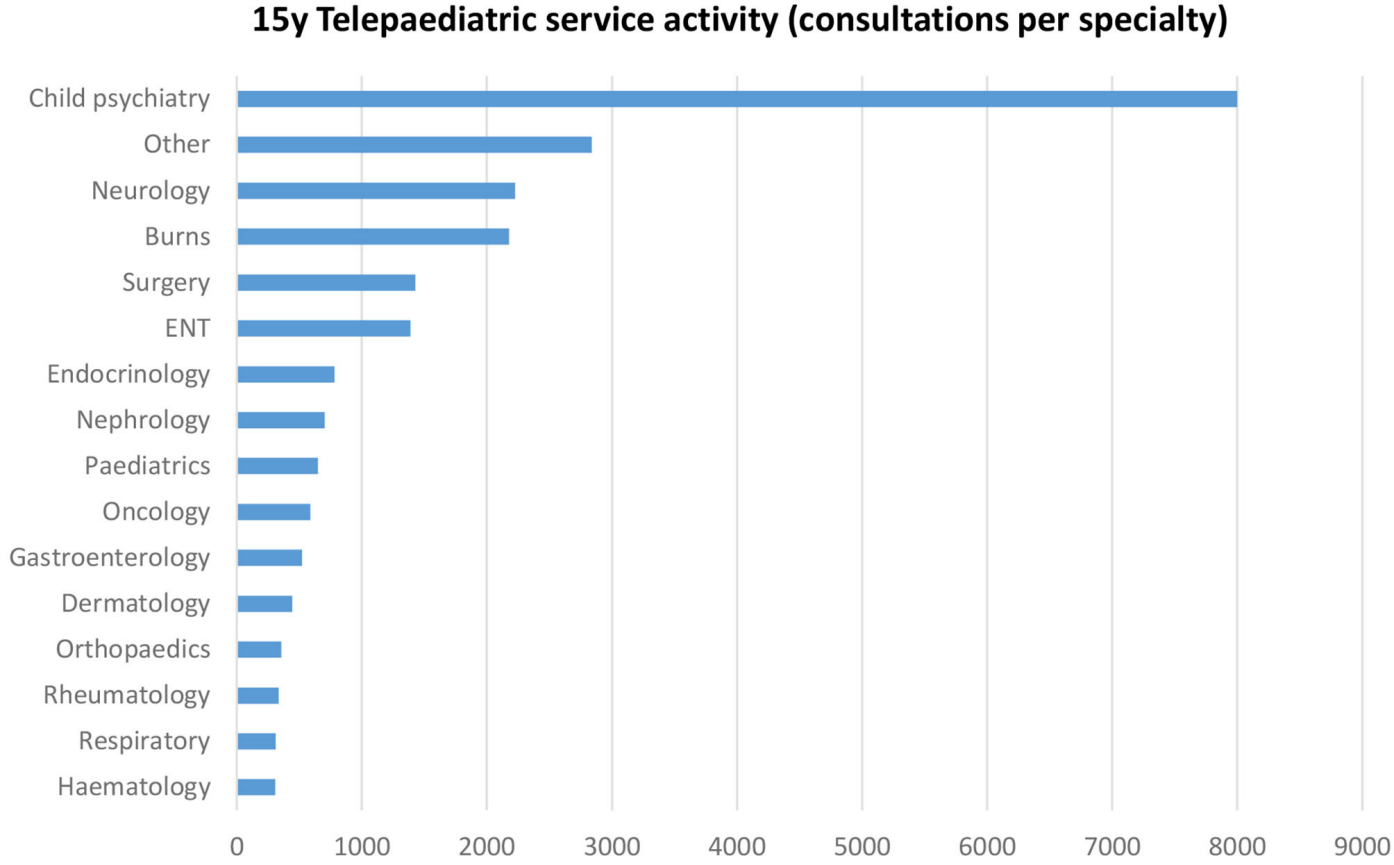
Number of QTS consultations per specialty, from November 2000 to March 2016.

The most common specialties were child psychiatry (35%), neurology (10%), burns (9%), surgery (6%), and Ear, Nose, and Throat (6%), see Figure 3. “Other” services included metabolic, cardiology, neurosurgery, palliative care, ophthalmology, immunology and allergy, neonatology, plastic surgery, dietetics, rehabilitation, speech pathology, pain management, audiology, occupational therapy, physiotherapy, infectious diseases, child development, and social work.

**Figure 4:**
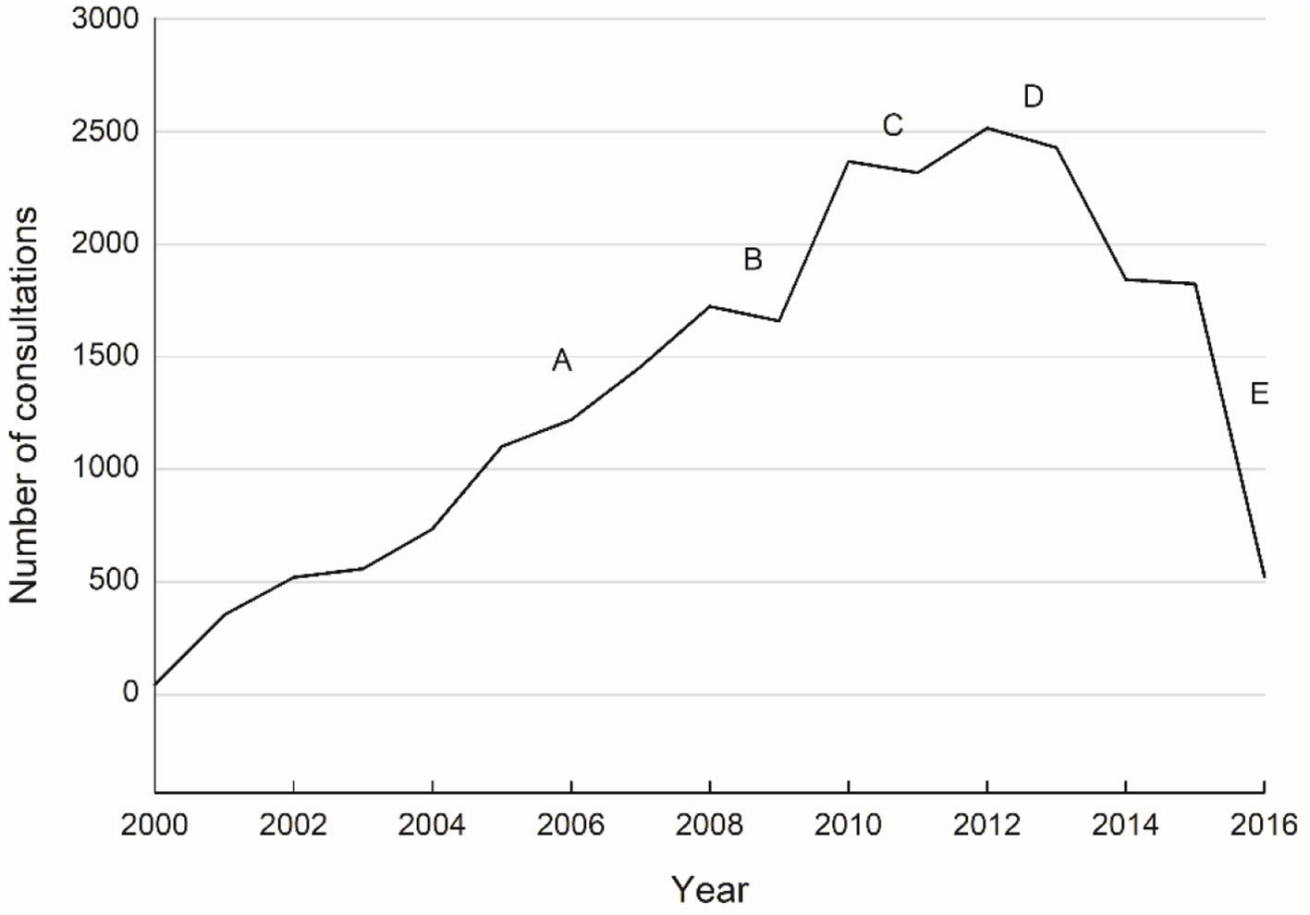
All QTS consultations over time (November 2000 to March 2016). Milestones: **(A)** mobile videoconferencing systems deployed for regional paediatric ward rounds; **(B)** mobile ENT surveillance service established; **(C)** QTS operational budget reduced; **(D)** staged closure of the RCH; and **(E)** transfer of the QTS to the new Queensland Children's Hospital in Brisbane.

The volume of telehealth activity gradually increased during the first 10 years (2000–2010), with the introduction of new specialties and expansion of services within certain clinical disciplines, see Figure 4. In 2006 (A), our mobile videoconferencing systems were used to provide pediatric support to regional hospitals (child-friendly robot ward rounds); and in 2008 (B), the mobile ear, nose and throat (ENT) surveillance service for Indigenous children was established in Cherbourg, resulting in additional ENT consultations at the RCH. From 2010 onwards, activity levels remained static or fell slightly. This mainly coincided with a significant reduction in the QTS operational budget (C); staged closure of the RCH (D); and the transfer of the QTS (E) over to the new children's hospital in Brisbane.

Referrals for telehealth consultations were made from over 270 health services, mainly hospitals throughout Queensland and northern New South Wales. The top five referring sites were Mackay (20%), Atherton (14%), Hervey Bay (7%), Mt Isa (5%), and Innisfail (5%). Almost all referrals originated from a regional hospital—or from the specialist hospital (provider site) for patient follow-up.

The QTS was primarily a clinical service; 97% of its use involved providing advice about a patient, reviewing a case, initial assessment before transfer to the specialist hospital or handover of a patient before return to regional hospital. The remaining activity (<3%) concerned the delivery of education or administrative services.

### Service Models

The QTS was flexible and responsive to clinical needs; a variety of different service models were developed amongst the specialist areas, reflecting the needs of the patient and the purpose of the consultation. Some were developed for general outpatient appointments with specialists, emergency advice for the assessment of infants with a cardiac condition, follow-up of patients receiving specialist burns care, case conferencing with regional health care teams involved in the care of children and families with mental health conditions, handover of patients to regional hospitals and home care, and community-based assessments of children with chronic ear disease. Other applications were primarily developed for education and training purposes.

#### General Outpatients

The most common application within the telehealth service was the delivery of outpatient appointments for children and families who would normally travel to Brisbane. Most clinical specialties were actively engaged and provided telehealth clinics on either a weekly or a monthly basis depending on demand. Common examples included clinics for diabetes, neurology, orthopedics, nephrology, rheumatology, and pediatric surgery (6–11). Often these clinics would be run in parallel with the in-person clinics in Brisbane, and the specialist time was allocated as required to the telehealth service. For certain specialties, a telehealth clinic list was established prior to the session, and connections involved multiple patients at the same (referring site) or multiple patients in a range of different sites. Telehealth clinics all required careful coordination to ensure site preparation and collection of the necessary clinic information in advance of the consultation.

#### Ad-hoc and Urgent Consultations

The assessment of newborn children with suspected cardiac defects was one of the services offered by the QTS. A pediatric cardiologist was able to assess infants remotely, by instructing the remote sonographer and viewing the echocardiogram in real-time (12). This meant a timely diagnosis and management plan could be discussed with the remote pediatrician caring for the child, and an informed decision could be made whether to transfer the infant to the specialist hospital or not. In the majority of cases, transfer of infants was then avoided and the infants continued to be managed locally with remote specialist support/advice as required. This service also provided the sonographer conducting the scan with valuable training experience whilst working with the cardiologist (13).

#### Post-acute Burns Care

In Queensland, specialist burns care is provided by one hospital. Referral guidelines for children with a serious burn injury indicate that referral to the specialist is necessary. Once care is received, follow-up care may last for many months or years. Prior to the use of telehealth, some outpatient appointments in Brisbane lasted for only minutes, despite some travel to the hospital taking many hours. The use of telehealth for outpatient burns care has revolutionized the support for children throughout the state (14). The burns team regularly provide videoconference appointments to all throughout Queensland and northern New South Wales. Appointments often involve occupational therapists (OTs) and nurses in regional hospitals, and the specialist burns staff in Brisbane (a medical consultant, OT and Nurse). In addition to the general follow-up appointments, telehealth has also been very useful for interim advice for a burn injury—to assist with immediate treatment at the remote hospital and planning for the transfer of the patient (15).

#### Mental Health Services

The delivery of telehealth by the e-child and youth mental health services (e-CYMHS) demonstrates a very effective model, combining conventional outreach services (where the specialist team travel to the regional towns) with telehealth support (16, 17). Most telehealth clinics involve case conferencing, where a series of cases are presented to the specialist team (psychiatrist and other mental health clinicians) via videoconference. In some cases, the patient's family would also participate in the session. As with the overall QTS, the success of the e-CYMHS was attributed to the role of the dedicated e-CYMHS telehealth coordinator. The telehealth component of the service was a very cost-effective way of keeping in contact with regional sites (see section on cost savings) (18).

#### Discharge Planning and Home Care

For patients receiving specialist care in Brisbane, telehealth was used to support the process of back-transfer to regional hospitals or to home care. In these cases, specialist teams could hand over important information about the cases via videoconference and ensure that regional staff and families were prepared for and understood the clinical care requirements. Anecdotally, families reported that they felt at ease knowing that the regional staff were familiar with the treatment and follow-up care. This was fairly common practice for children referred from the oncology and palliative care unit (19, 20). In the case of home care, often the home nursing services and local general practitioners were engaged in the telehealth service.

#### Community-Based Health Surveillance

**Figure 5:**
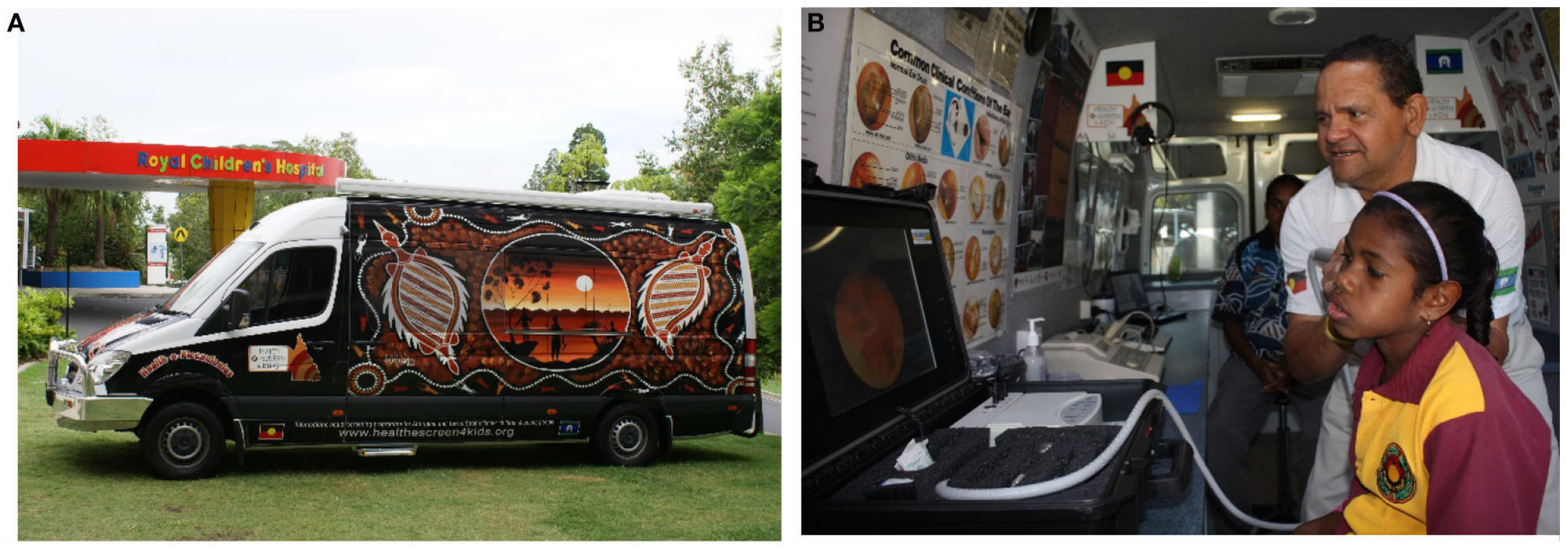
**(A)** Mobile screening van used for surveillance of Aboriginal and Torres Strait Islander children at risk of ear disease and **(B)** child being screened by an experienced Aboriginal Health Worker.

Through consultation with the Cherbourg community health service (~260 km from Brisbane) and specialists in Brisbane, we developed a surveillance program for Aboriginal and Torres Strait Islander children at risk of ear disease, to ensure early detection and referral for treatment. We developed a mobile telehealth-enabled ear screening service, see Figure 5, which was operated by an experienced local Aboriginal Health Worker (AHW) (21). The AHW used the mobile service to visit schools and routinely assess Indigenous children. The AHW assessments included pure-tone audiometry, tympanometry, and digital otoscopy. In cases where children failed a screening test or if the AHW had any concerns, the AHW assessments were shared asynchronously via a secure online database, and reviewed by an ENT specialist. Assessment and treatment planning would then be done by the specialist and/or referred to the local medical service. This screening service has resulted in improvements in overall screening rates and the emergence of a model of care, which is community led and culturally appropriate (22, 23).

#### Education and Training

Whilst the majority of services delivered through the QTS were of a clinical nature, the use of videoconferencing was also important for education and training purposes. All clinical consultations had an intrinsic educational benefit because of the interaction that occurred between specialists and clinicians at the referring sites. Anecdotally, clinicians appreciated the service because of the learning opportunities it offered. Specific services were also developed to support the training requirements of regional staff responsible for children with special care needs (such as burns care and child development). When patients were being directed back to primary and secondary centres, it was important that clinicians were supported with clinical education (24, 25). Students undertaking their clinical training in rural and remote hospitals were also supported by the QTS, with access to interactive lectures by videoconference—allowing participation irrespective of location (26).

### Cost Savings

The majority of savings were associated with the reduced need for patient travel. Economic evaluations using cost-minimization analysis methods demonstrated the level of activity required to reach a threshold, whereby the costs of providing one service were the same as the other. The child and youth mental health service, which was responsible for almost one-third of all QTS activity, demonstrated that at the level of activity achieved in their service, it was less expensive to provide telehealth services than doing outreach (where the specialist team traveled to the regional town) or arranging for the patient and family to travel (18, 27). Similar studies showed potential savings to the health service for ENT services (28).

### Key Enablers

A key factor in the success of this program was the centralization of support made available by the QTS, which made the referral, consultation and documentation process convenient for clinicians. Another key factor was the integration of telehealth services on a business as usual basis, which was reflected in clinic schedules, service delivery planning and staffing allocations for each specialty. Running these clinical activities alongside a robust research program gave clinical teams the opportunity to contribute to the evaluation process and also to the planning of innovative services within the department. Clinician availability and support for both the near (provider) and far (receiver) end was very important—as was the need to train clinicians in certain skills relevant to telehealth consultation processes. The telehealth process also required new referral processes—and once these were made clear, the coordination of appointments and clinics became more straightforward.

Access to high quality telehealth facilities in a central and easily accessible location was important. The COH provided dedicated telehealth studios, which were used for most clinics. Over time, with improvements in software-based videoconferencing systems, some clinical groups were able to conduct their own telehealth work within their own department. This still required an appropriate place, which was private, and had good lighting and suitable acoustics.

### World Firsts

Academically, the COH published over 75 journal articles relating to “telepaediatrics” during the 15-year period; and pioneered a number of “world firsts” which are improving access to health and support services for regional families. These included the establishment of QTS—the first fully serviced multidisciplinary pediatric telehealth service (2, 3); the first child-friendly mobile telehealth service (robots) (5, 24, 29); the first use of telehealth for the delivery of clown doctor outreach services (30); and the first telehealth-supported Indigenous ear screening service with online links to pediatric specialists (21, 22).

### Challenges

Funding to cover the cost of telehealth is a commonly reported challenge, and one faced by the QTS since establishment. Initially, QTS telehealth services were not funded, so unless the telehealth service was purely substitution of face-to-face clinic appointments, then clinicians were providing services without direct funding. In 2011–2012, new funding opportunities emerged when the Commonwealth Government introduced funding for specialist video consultations under the Medical Benefits Schedule (MBS) (31). Around the same time, the Queensland Government Statewide Telehealth Unit introduced incentive funding for telehealth, to promote the uptake of telehealth. This incentive funding was in addition to activity-based funding which includes all activity (telehealth and in-person consultations). In small rural hospitals, activity-based funding is typically not viable due to relatively lower activity, and therefore block-funding arrangements are supported by the health department. In 2020, new temporary funding was introduced by the Australian Government in response to the 2019 coronavirus pandemic (COVID-19). Collectively, these funding developments have resulted in substantial growth in telehealth activity across Queensland and throughout Australia (32, 33).

Staff availability was also a challenge because telehealth sessions not only relied on the availability of the specialist, but also the availability of the referring clinicians and support staff at the regional hospital. We addressed this challenge by setting up clinics in advance, so that regular clinical days and times were available—either on a weekly, fortnightly, monthly or quarterly basis, depending on demand. The delivery of telehealth also changed from an *ad-hoc* arrangement to an appreciation that telehealth was integrated and routine. Like any telehealth operation, we did experience some staff resistance, but this was mainly related to the lack of clear processes, time constraints, and telehealth awareness. In this context, clinician acceptance and willingness to practice were important factors in the uptake of telehealth (34). Resistance transformed into interest over time as clinicians gained experience, and processes were put into place to ensure appropriateness of telehealth referrals and case preparation (case history and other relevant documentation) (35).

## Discussion

The QTS represents an extensive program of work conducted over a 15-year period. Examples of pediatric telehealth services have emerged as a result of different clinical requirements. The expansion of telehealth was sustained over an extensive period of time, and for a small number of mature services, we observed a willingness to conduct telehealth consultations outside of the telehealth centre and in the clinical departments. This worked particularly well when there was administrative assistance available in the department to help prepare cases, conduct test calls, send appointment details to families and help with the documentation (hospital records, investigative tests, referral notes etc.). The work done in Queensland also highlighted the importance of the role of a telehealth coordinator. This was a key requirement for the facilitation of services and an intended strategy to ensure that the referral and telehealth consultation processes were managed efficiently and without unreasonable burden on the clinician. Originally considered a superfluous resource by some health managers in Queensland, telehealth coordinator positions are now fully supported throughout the state—and recurrently funded by the health department, on a business as usual basis.

Over time, it was encouraging to see the number of specialties engaged in the QTS. It was clear that telehealth was and has continued to be used as a routine method of consultation for medical, nursing and allied health staff in the health service. The development of the service was also inspired by a variety of COH-led research projects, which helped to generate new ideas amongst clinicians when caring for children and families in remote locations. Funding for these projects was mainly derived from competitive research grants and philanthropic funding. Combining research and service delivery was a useful process because it meant that clinician engagement was strong and ideas were generated in direct response to clinical needs. The duration of the service development work also meant that information could be collected to demonstrate trends in activity and opportunities for service growth.

The work highlighted in Queensland is one of the most prominent examples of telepaediatrics reported worldwide, operating over a significant period and demonstrating a large volume of activity across many different specialties. Other successful examples exist in the USA and Canada where telehealth services have been established for emergency and intensive care support, hospital outpatients, primary care and home support (36–41). Work in California also demonstrated cost savings and significant environmental benefits due to reduced travel requirements for patients—hence another reason for doing telehealth (42).

### Key Lessons Learned

1. The establishment of a successful telehealth service requires time, patience and close engagement with clinicians and health service managers.
2. Effective telehealth services require dedicated administrative support services (telehealth coordination) and strong clinical leadership.
3. Integrating telehealth into existing hospital systems (such as referral and triage management, scheduling and billing processes) is important for services to become routinely adopted.
4. Clinician involvement in the planning and delivery of new telehealth-supported models of care ensures that services fundamentally address clinical requirements and patient needs.
5. The broad nature of services established through the QTS demonstrates the value of telehealth for a diverse range of clinical specialities and also highlights the importance of different service models for different clinical areas (not a one-model-fits-all approach).
6. Most potential savings attributed to the QTS were related to reduced patient travel.
7. The unique partnership between the COH (university) and the health department (service) resulted in 15 years of pioneering work and the development of a rich evidence base for telepaediatrics.

## Conclusion

The QTS work has laid the foundations for the provision of pediatric telehealth services in Queensland, and many of the service models have been replicated in other places throughout Australia. During the operational period reported in this review, the partnership between the service provider and the university was a unique opportunity to leverage research funding and to drive innovation within the service. We encourage future reviews of the QTS to monitor progress and to demonstrate the benefits for children and families living in remote locations. It is highly likely that new telehealth-enabled models of care will continue to evolve in response to the many challenges faced by the health department, new funding arrangements, advances in communications technology and the expectations of consumers due to increased experience and raised awareness of telehealth.

## Data Availability Statement

The raw data supporting the conclusions of this article is available upon request, subject to ethics approval requirements.

## Ethics Statement

Written informed consent was obtained from the individual(s), and minor(s)' legal guardian/next of kin, for the publication of any potentially identifiable images or data included in this article.

## Author Contributions

AS was responsible for establishing the QTS and for leading the establishment of most of the services described in the review, obtaining clearance from the appropriate ethics committee, leading the analysis of activity, and drafting the manuscript with input from all authors. AS, NA, and LC conceived and designed the review of the QTS and were responsible for data collection. All authors were involved in reviewing the manuscript and critically appraising the content and were responsible for final approval of the manuscript before submission for publication.

## Conflict of Interest

The authors declare that the research was conducted in the absence of any commercial or financial relationships that could be construed as a potential conflict of interest.
